# Consideration of Social Disadvantages for Understanding and Preventing Obesity in Children

**DOI:** 10.3389/fpubh.2020.00423

**Published:** 2020-08-28

**Authors:** Alelí M. Ayala-Marín, Isabel Iguacel, Pilar De Miguel-Etayo, Luis A. Moreno

**Affiliations:** ^1^Growth, Exercise, Nutrition and Development (GENUD) Research Group, Universidad de Zaragoza, Zaragoza, España; ^2^Instituto de Investigación Sanitaria Aragón (IIS Aragón), Universidad de Zaragoza, Zaragoza, España; ^3^Instituto Agroalimentario de Aragón, Universidad de Zaragoza, Zaragoza, España; ^4^Departamento de Fisiatría y Enfermería, Universidad de Zaragoza, Zaragoza, España; ^5^Centro de Investigación Biomédica en Red de Fisiopatología de la Obesidad y Nutrición (CIBERObn), Instituto de Salud Carlos III, Madrid, España

**Keywords:** social disadvantages, socioeconomic status, children, obesity determinants, socio-ecological model, multilevel interventions

## Abstract

Addressing social disadvantages that lead to obesity should be a public health priority. Obesity prevalence among children and adolescents has reached a plateau in countries with high income but it continues rising in low-income and middle-income countries. In high-income countries, an elevated prevalence of obesity is found among racial and ethnic minority groups and individuals from disadvantaged socioeconomic backgrounds. In addition to classic socioeconomic status (SES) factors, like income, parental education, and occupation, recent publications have linked parental social disadvantages, such as minimal social network, non-traditional family structure, migrant status and unemployment, with obesogenic behaviors and obesity among children. Socio-ecological models of obesity in children can explain the influence of classic SES factors, social disadvantages, culture, and genes on behaviors that could lead to obesity, contributing to the elevated prevalence of obesity. Obesity is a multifactorial disease in which multilevel interventions seem to be the most effective approach to prevent obesity in children, but previous meta-analyses have found that multilevel interventions had poor or inconsistent results. Despite these results, some multilevel interventions addressing specific disadvantaged social groups have shown beneficial effects on children's weight and energy balance-related behaviors, while other interventions have benefited children from both disadvantaged and non-disadvantaged backgrounds. Considering obesity as a worldwide problem, the World Health Organization, the European Commission, and the National Institutes of Health recommend the implementation of obesity prevention programs, but the implementation of such programs without taking into consideration social disadvantages may be an unsuccessful approach. Therefore, the present publication consists of a review of the pertinent literature related to social disadvantage and its consequences for behaviors that could lead to childhood obesity. In addition, we will discuss the relationship between social disadvantages and the socio-ecological model of obesity in children. Finally, we will summarize the relevant aspects of multilevel intervention programs aiming to prevent obesity in children and provide recommendations for future research and intervention approaches to improve weight status in children with social disadvantages.

## Introduction

Addressing social disadvantages that lead to overweight and obesity should be a public health priority. In 2016, the global prevalence of children under 5 years old and children/adolescents aged 5–19 years old with overweight/obesity was estimated to be over 38 and 340 million, respectively (1). Obesity prevalence among children and adolescents has reached a plateau in countries with high income but it continues rising in low- and middle-income countries (2–4). The lack of representation in the global prevalence studies of subgroups with low socioeconomic status (SES) can explain the plateau in the prevalence of obesity among high-income countries (2, 3). Despite this plateau, to date, the prevalence of overweight/obesity for high-income countries is high, while the prevalence among low and middle-income countries is increasing (3, 4).

Obesity in children can lead to overweight or obesity in adulthood. Also, childhood obesity is associated with health problems in adulthood such as insulin resistance, type 2 diabetes, hypertension or hyperlipidemia, and psychological problems like depression and eating disorders (5). As a result, in adulthood, the treatment of overweight or obesity and the chronic diseases that coexist with them represent high healthcare utilization. Among high-income countries, people with obesity have ~30% more medical costs than people with normal weight (6). Similarly, adults living in Spain with a body mass index (BMI) ≥35 kg/m^2^ use approximately twice as many primary care, home care, and psychology visits than adults with a normal BMI (7). In the United States (US), a country in which the healthcare system is based mainly on private insurance and out-of-pocket payments, the estimated yearly cost of medical visits, prescription drugs, or home care visits related to obesity is $149.4 billion (8). The costs of obesity are related to high healthcare utilization or medical costs and also to the cost of early retirement, disability, and early mortality. From 1990 to 2010, the global deaths (years of life lost) and disability (years living with disability) attributable to a high BMI increased from 52 to 94 million years (9). A high BMI is the main risk factor for deaths and disability in Australasia and southern Latin America (9). A study from Germany reported that the obesity costs associated with sick leave, early retirement, unemployment (short- and long-term), and early mortality were higher than the medical, nursing, and rehabilitation costs (€33.65 vs. €29.39 billion, respectively) (10). The health care costs and the productivity loss and early mortality due to obesity are elevated among high-income countries which have more resources to implement strategies to prevent it in comparison with middle- and low-income countries.

Most of the interventions implemented to prevent obesity in children have been conducted in high-income or developed countries (11, 12). Among high-income countries, a high prevalence of obesity and obesogenic behaviors is observed in children and adults from racial or ethnic minorities or socioeconomically disadvantaged backgrounds (13–15). This is especially relevant in the design and implementation of interventions aiming to prevent obesity. According to the behavioral change theory, intervention favors subgroups without disadvantages as they have more resources to achieve behavioral change (16). Few intervention studies have analyzed their effectiveness by SES or by disadvantaged groups (12). Consequently, there is no sufficient evidence on which intervention or strategies are effective to prevent obesity in children and adolescents with social disadvantages (17–19).

The cause of obesity is mostly described as an imbalance between the calories ingested and the calories expended. Diet, physical activity, and sedentarism are behaviors that impact positively or negatively on weight status. Apart from behaviors, there may be other factors that set their influence over obesogenic behaviors or weight status. Recently, classic SES indicators like income, parental education, and occupation, as well as parental social disadvantages, such as minimal social network, non-traditional family structure, migrant status, and unemployment were associated with obesogenic behaviors and obesity among children (20–28). To this date, the data available relating to these factors with obesity do not demonstrate a causal relationship, but the association results can explain why in some prevalence studies a high prevalence of obesity is observed among children and adults from disadvantaged backgrounds and from minority ethnic/racial subgroups (29–31). A better understanding of the complex relationships between biological, cultural, and socioeconomic determinants that can lead to overweight or obesity will facilitate the design of effective strategies to prevent it.

Therefore, the present publication is a review of the pertinent literature related to social disadvantage and its consequences for behaviors that could lead to overweight/obesity status in children. We will discuss the relationship between social disadvantages and obesity in children with a special focus on SES classic indicators and social disadvantages recently defined in the literature. We will examine the socio-ecological model of obesity in children described in the scientific literature. Finally, we will summarize the relevant aspects of multilevel intervention programs aiming to prevent obesity in children and provide recommendations for future research and intervention approaches to improve weight status in children with social disadvantages.

## Classic Ses and Social Disadvantages and Their Consequences in Obesogenic Behaviors, Obesity, and Other Health Conditions

Socioeconomic disadvantages have been defined as social or economic conditions or events that may negatively affect children (32, 33). These stressors can be social conditions (such as being a migrant) or economic situations (such as belonging to a low-income family) and might occur at different levels (individual, household, community, or society level). In this section, we will introduce social disadvantages and how they relate to weight and to the most relevant obesogenic behaviors in children.

Traditionally, socioeconomic disadvantages have been related to classic SES indicators (low education, low occupation, and low income). Socioeconomic disadvantages can affect child weight status as early as in the conception stage. Pregnant women with low education are more likely to have a gestational weight gain above the Institute of Medicine recommendations, a risk factor for future overweight in children (34). Low educational attainment is not the only socioeconomic factor related to the gestational weight gain. A study from the US observed that pregnant women with college education that lived in a medium- or low-SES neighborhood or that had a Hispanic background gained more gestational weight than women with less education (35).

In the first years of life, social disadvantages can interfere with breastfeeding, a protective factor against obesity in children (36). Lower SES and the presence of social disadvantages prevent the adoption and the continuation of breastfeeding as a source of exclusive nutrition during the first 6 months of life. In developing countries, lower educational attainment and a non-traditional family structure have been reported as barriers to exclusive breastfeeding (37). Among employed women, a shorter maternity leave, maternal full-time employment, and the lack of breastfeeding support in the workplace, either in developing or developed countries, were acknowledged as obstacles for breastfeeding (37, 38).

In high-income countries, school-aged children whose parents have low SES are more likely to adopt unhealthy dietary patterns (39). Physical activity and, especially, sedentary behaviors in children have been linked to classic SES indicators. Children with low-educated, low-income, or low-occupation parents tend to report less physical activity (although literature has yielded mixed results) but, above all, more screen time (such as videogames or TV) (40). One of the reasons behind this finding might be the fact that children from low-SES families are less likely to be sport club members compared to higher SES families (21). In developed and developing countries, these unhealthy dietary patterns plus higher levels of sedentarism observed in children from low-SES backgrounds result in higher rates of overweight and obesity (41). There are several mechanisms that have been argued to explain these harmful behaviors that go beyond the mere economic fact. The consumption of unhealthy foods in youth can be the result not only of affordable options for these families but also of combinations of multiple factors. In fact, families from low-SES backgrounds are more likely to live in neighborhoods with abundant sources of foods that promote unhealthy eating (42). Having limited access to supermarkets and grocery stores can be a significant barrier to the consumption of healthy foods. Also, unsafe neighborhoods can be an obstacle to physical activity (43, 44).

Socioeconomic disadvantages include not only these classic SES indicators but also social vulnerabilities that go beyond this term. A number of social vulnerabilities during childhood and adolescence have been examined in recent papers (migrant status, lack of a social network, parental unemployment, teenage pregnancy, not living with two biological parents, or parental substance use) (33, 45, 46). Although the possible effect of these social vulnerabilities in children might be moderated by classic SES indicators, these social vulnerabilities have an independent effect since they are stressors *per se*. For example, having a migrant background or the lack of a social network can be a vulnerable situation with a direct psychological effect independent of education, occupation, and income (46).

The strong and inverse relationship between obesity and SES in high-income countries can be explained by different mechanisms. Socioeconomic disadvantages have been linked to psychosocial problems, and several investigations have found an association between psychosocial well-being and overweight (47, 48). In fact, a scientific paper that investigated the associations between socioeconomic disadvantages and psychosocial problems found that having parents with a lack of a social network was the strongest determining factor to predict children's internalizing and psychosocial problems (46). The negative impact of childhood overweight on psychosocial well-being has been demonstrated in many studies, but the association between overweight and psychosocial well-being may be bidirectional because there is also evidence that psychosocial well-being may influence future overweight (49).

The stressful experiences (derived for example from the instability and uncertainty of unemployment) may be cardiometabolic risk factors for parents and children not only through lifestyle factors (such as sedentary behaviors, unhealthy diet, or the consumption of drugs by children and adolescents) but also through direct physiological changes due to the alteration of regulatory pathways. This alteration comprises the activation of the hypothalamic pituitary adrenal axis, the sympathetic nervous system, and other systems as a response to stress derived from these vulnerable situations. The maintained activation of these systems over time can lead to a cascade of physiological processes such as the rise of cortisol, glucose, or inflammatory markers (50).

Children with socioeconomic disadvantages therefore might be more likely to have metabolic disorders than children without disadvantages. Risk factors for cardiovascular diseases and diabetes include abdominal obesity, hypertension, insulin resistance (IR), elevated triglycerides (TG), and reduced high-density lipoprotein cholesterol (HDL-C) tending to cluster as a metabolic syndrome. Several diagnostic criteria for metabolic syndrome have been proposed. A harmonized definition of metabolic syndrome was established in 2009 with at least 3 or more criteria required for diagnosis: elevated waist circumference (population- and country-specific definition), blood pressure over 130/85 mmHg, fasting triglyceride (TG) level over 150 mg/dl, fasting high-density lipoprotein (HDL) cholesterol level <40 mg/dl (men), or 50 mg/dl (women), and fasting blood sugar over 100 mg/dl (51). All definitions of metabolic syndrome in children are closely related to the presence of overweight and obesity, which is consistent with the high prevalence of metabolic syndrome observed among prepubertal children and adolescents with obesity (52, 53). As children with social vulnerabilities present higher rates of obesity than children without social disadvantages, literature has often linked these high rates of obesity with high rates of metabolic syndrome presented in vulnerable groups. Nevertheless, as indicated before, this would not be the unique path that would explain the high rates of metabolic disorders presented by vulnerable children. One of the factors that seem to mediate all these relationships is stress derived from these most unfavorable situations, and various articles have linked the stress maintained over time with obesity and metabolic syndrome. Disadvantaged groups may be more exposed to chronic stress, due, among others, to their social isolation, financial limitations, and lack of social support, which could lead to negative feelings and behaviors in the parents and create a stressful context in the lives of children. Stress can produce adaptive changes in the metabolism in the short term, but stress responses can become maladaptive when the organism is under long-term stress (54, 55). The reasons that could explain why metabolic syndrome is more common among socioeconomically disadvantaged groups compared to groups without disadvantages are based on mental, biological, and behavioral health factors (56). Socioeconomic disadvantages increase the risk of mood and anxiety disorders, which are mental health factors that increase cardiovascular morbidity and mortality. Socioeconomic disadvantages can disrupt many of the systems of the body by altering the immune, metabolic, neuroendocrine, and autonomous nervous system. Finally, as previously said, socioeconomic disadvantages are related with adverse behavioral factors such as smoking, physical inactivity, and a higher intake of energy-dense foods that increase cardiovascular diseases.

## Socio-Ecological Models of Obesity in Children

Socioeconomic disadvantages do not occur in isolation. For example, it is possible that parents who have a lower educational attainment will have a low occupational level which translates into earning a low income. These parents will possibly live in a low-SES neighborhood which is characterized by being unsafe, and by lacking a built-environment (side-walks or areas for recreation) or markets with whole grain products, fresh fruit, or vegetables. Therefore, these parents and their children will not have the individual, household, community, or social resources to engage in a healthy lifestyle. The Socio-Ecological Model (SEM) is a theory-based framework inspired by Bronfenbrenner's ecological systems theory that describes the environmental influences structures (or levels) and their interaction in the development of a person (57). SEMs have been addressed by many authors but to our knowledge there are only four review articles that comprehensively describe the complex relationship between multiple factors and the occurrence of obesity in children (Table 1). All the authors acknowledge that a variety of determinants, which can be grouped into levels of influence, are related to the occurrence and persistence of obesity in children (58–61).

**Table 1:** Comparison of the levels and the determinants related to classic SES and social disadvantages described in the published Socio-Ecological Models (SEMs) of obesity in children.

**Author, year**	**SEMs characteristics**	**Levels (bold) and classic SES**^****a****^ **and social disadvantages**^****b****^ **determinants** ***(italics)*** **from the inner to the outer layer**					
		**Inner level**					**Outer level**
Davison and Birch (58)	Population: children (country not specified) Total levels: 4 Relationships: Bidirectional relationship between levels	**Child weight status**	**Child characteristics and child risk factors**	**Parenting styles and family characteristics**	**Community, demographic and societal characteristics** •*SES* •*Ethnicity* •*Work demands*	–	–
Harrison et al. (59)	Population: infants to adolescents (country not specified) Total levels: 6 Relationships: Bidirectional relationship between and within levels. The influence of levels over the individual changes over time	**Cell (child gene and biological characteristics)**	**Child (child characteristics)** •*Race/ethnicity*	**Clan (family characteristics)** •*SES* •*Race/ethnicity* •*Parent work schedule*	**Community (local community or organizational characteristics)** •*SES* •*Race/ethnicity* •*Local employment practices* •*Social marginalization*	**Country (state and national characteristics)**	**Culture** **(cultural and societal characteristics)**
Willows et al. (60)	Population: aboriginal children living in Canada Total levels: 6 Relationships: Bidirectional relationship between and within levels. The influence of levels over the individual changes over time	**Individual** •*Ethnicity*	**Interpersonal (family and peers)** •*Peer and family support*	**Community, home and sociocultural environments** •*Household SES* •*Family structure*	**Built environment**	**Society**	**Historical factors** •*Colonization* •*Assimilation policies*
Zhou and Cheah (61)	Population: Chinese immigrant children living in United States Total levels: 5 Relationships: Bidirectional relationship between and within levels. The influence of levels over the individual changes over time	**Child** •*Migrant origin*	**Microsystem (family, peers, and health services)** •*Migrant origin*	**Mesosystem (utilization of resources)** •*Migrant origin*	**Exosystem (community and occupation)** •*Parental occupation* •*Community SES* •*Migrant origin*	**Macrosystem (social attitudes and cultural ethno-theories)** •*Migrant origin*	–

a *Classic SES indicators are the determinants related to education, income and occupation*.

b *Social disadvantages are the determinants related to lack of family support, non-traditional family, migrant background (or minority racial/ethnic background), and lack of employment*.

Bronfenbrenner describes the inner level of influence as the core of the individual which is followed by a layer related to the immediate setting (such as the home or classroom) and a layer with the external environment aspects (57). The SEMs evaluated share these characteristics. We observe that the core of the SEMs is the child's biological or behavioral aspects, followed by the levels of influence related to the social and environmental aspects from the family and school levels, and ending with the community and society aspects as the outer layers of the models (58–61).

The SEMs included in the present review slightly differ in their levels. The differences found were relating to the demographical characteristics of the population for which the SEMs were developed. For example, the SEM of obesity for aboriginal children living in Canada has an additional layer of historical factors (colonization by Europeans, dispossession of traditional lands and assimilation policies) which it is not observed in the other three SEMs (60). Willows et al. recognizes that historical factors have undermined aboriginal cultural values such as agriculture, healthy food choices, and adequate physical activity. The differences between levels of the SEMs are consistent with Bronfenbrenner's theory. This theory explains that even though some levels are shared between groups, the influence of those levels is different for subgroups of a given society (57). Despite the differences between levels, it can be observed that all the authors include classic SES determinants in the SEMs as well as factors related to race/ethnic minority and migrant background (58–61).

The SEM of obesity in children is complex. The relationship of the determinants is between and within each level and they are related to other determinants that are not social disadvantages like behaviors, parenting styles, culture, and stress. Finally, the influence of the determinants over the individual changes over time (59–61). It is important to point out, that due to the complexity of the obesity determinants, the authors included the most relevant childhood obesity determinants that will contribute to the development of future obesity preventive strategies (58–61). In Table 1, we focus specifically on how these authors have incorporated classic SES and social disadvantage as determinants of obesity in their SEMs.

## Multilevel Intervention Programs Aiming to Prevent Obesity in Children With Disadvantages

SEMs of obesity in children explain the influence of classic SES factors, social disadvantages, culture, and genes on behaviors that could lead to obesity. The World Health Organization, the European Commission, and the National Institutes of Health recommend the implementation of multi-sectorial and multi-component obesity prevention programs admitting that no single action will halt obesity (62–64). Multilevel interventions or interventions that address multiple levels of influence (as suggested in the SEMs) seem to be the most effective approach to prevent obesity in children. However, previous meta-analysis found that multilevel interventions had poor or inconsistent results (18, 65). Some multilevel interventions addressing specific disadvantaged social groups have shown beneficial effects on children's weight and energy balance-related behaviors, while other interventions have benefited children from both disadvantaged and non-disadvantaged backgrounds (19).

Few studies have analyzed the relationship between disadvantages and the effectiveness of interventions aiming to prevent obesity. Robl et al. analyzed the influence of individual and cumulative social disadvantages (migrant background, attendance at a school for children with special needs and parental unemployment) on a multidimensional lifestyle intervention implemented in Germany, Austria and Switzerland (66). The intervention intended to improve weight among children and adolescents with obesity and the intervention included the following strategies: nutritional counseling, exercise, implementation of behavior modification, and parental training. Children without a migrant background, attending regular schools and with at least one fully employed parent had a greater reduction in BMI when compared with children with social disadvantages (children with parents working part-time or with no parents working full-time, and with both parents unemployed) (66). This result follows behavioral change theory which suggests that intervention favors groups without social disadvantages as they have more resources to achieve behavioral change (16). A slightly different result was observed in a multisector intervention study to prevent obesity by improving energy balance-related behaviors among children aged 2–4, participants of the WIC (Women, Infants, Children) program in US (67). The WIC program is a federal program which provides nutritional education, health care referrals, and supplemental foods for low-income families. The Massachusetts Childhood Obesity Research Demonstration (MA-CORD) intervention provided training sessions to the WIC program providers on obesity prevention counseling strategies to help participants improve their energy-balance behaviors. The intervention encouraged the intake of fruits and vegetables and promoted physical activity and adequate hours of sleep. MA-CORD intervention aimed to eliminate the intake of sugar-beverages, reduce the intake of 100% juices, and limit screen time (68). Children from minority ethnic/racial background that received the intervention decreased their BMI when compared to children from minority ethnic/racial backgrounds that had not received the intervention (67).

Some interventions have aimed to prevent obesity in children living in low-SES communities. A school-based intervention implemented in kindergartens pertaining to Arab-Israeli low-SES communities in Israel had short- and long-term beneficial effects on children. The intervention included a nutritional educational program and physical activity sessions (45 min per day for 6 days a week) which was offered by kindergarten teachers (69). The intervention involved the children's families. Parents received a monthly flier and were encouraged to discuss the information with their children. Both children and parents were invited to participate in health festivals. Children that received the intervention decreased their BMI and increased their nutritional and physical activity knowledge, improved their fitness, and had better nutritional and fitness preferences in comparison with the control group (69).

An intervention that improved the anthropometric measurements of children from low-SES groups in comparison with children from high-SES groups was the *Fleurbaix–Laventie Ville Sante* (FLVS) intervention (70). The FLVS intervention was a longitudinal study implemented in two towns in France (*Fleurbaix* and *Laventie*). The intervention was originally designed as a school-based nutritional program. Thanks to community engagement, the program transitioned into a multilevel intervention to sustain the nutritional program and to include environmental and community initiatives that lead to the employment of dietitians and sports educators, the delivery of community activities, and the construction of sports facilities (70). The FLVS intervention reduced the prevalence of obesity in the community in a 12-year period, and the prevalence of obesity was lower in children with parents from intermediate- or low-occupation levels.

The EPHE [*Ensemble Prévenons l'Obésité Des Enfants* (EPODE) for the Promotion of Health Equity] intervention study evaluated the effectiveness of an intervention in reducing SES inequalities associated with obesogenic behaviors in communities from seven European countries (Belgium, Bulgaria, France, Greece, Netherlands, Portugal, and Romania) (71). Children aged 6–8 years old and their parents participated in an intervention tailored according to the inequality gaps of the communities. Intervention outcomes in low-SES participants differed by community (country). Researchers observed an increase in the frequency of fruit consumption in the Netherlands, a reduction in the consumption of fruit juices in Romania, a reduction of the weekday hours of watching TV, and an increase of parental monitoring on a child's TV time in Belgium (71).

## Recommendations for Future Approaches for Obesity Prevention

The present review has demonstrated that children with low SES or with social disadvantages are more likely to engage in obesogenic behaviors and to weigh more than children with high SES or without social disadvantages. The complex relationships between and within the levels and determinants of obesity make it difficult to resolve the onset or to reverse current trends of obesity. In order to halt the obesity epidemic, a variety of integrated strategies need to be implemented. Recommendations to address all determinants associated with obesity in children are beyond the scope of this review. We will focus our recommendations on the “how to” establish obesity prevention priorities according to the community and society setting and on the “whom to” involve in the strategies.

### Understanding the Big Picture

We observed similar determinants across all the SEMs evaluated as part of this review, especially those related to the SES indicators and social disadvantages and their association with obesity. Social differences among countries and within communities may account for different multilevel strategies to intervene in the prevention of obesity. SEMs of obesity can be used as a tool to understand the different determinants that influence the onset of obesity and also to identify new determinants in a specific setting. Davison et al. combined quantitative and qualitative methodologies to validate a Family Ecological Model (FEM) of obesity in children previous to the design of a family-centered intervention (72). The FEM is comparable to the SEM, and the difference between both is that the FEM has the family as the focal point of the model rather than the individual (child). The study was conducted in a medically underserved community in the US, and the parents were participants of the Head Start Program. Head Start is a program that promotes school readiness of children from low-income families. The mixed-methods study methodology included surveys and interviews, focus groups, and photo-voice methodology in which parents documented the family and community factors that caused them stress. Also, the parents were asked about their social, economic, and community environment while driving through the neighborhood (72). The validation process of the FEM provided a better understanding of the complexity of obesity, especially among those with low SES within the family and at the community level. Most notably, it contributed to identify family assets such as having older siblings, to prevent obesity and to draft a logic model for an intervention that would take into consideration the ecology and the social and parenting practices of the families (72). Therefore, applying the SEM with methodologies that combine quantitative data from the researchers and qualitative data from the community can help to establish the prevention priorities according to the community and country setting.

### The Levels to Act and the Actors at Play

Despite the insufficient scientific evidence about multilevel interventions and their effectiveness by SES group, some researchers have identified prevention strategies that could possibly prevent obesity, improve weight, or improve behaviors in children and adolescents from disadvantaged backgrounds. Researchers agree that obesity can be prevented with a multilevel approach because it takes into consideration the levels of obesity determinants. Some interventions have used the whole-of-community (WOC) approach, or a geographic-specific multilevel intervention approach to prevent obesity among children (19). A systematic review of WOC interventions implemented in high-income countries found that 10 interventions had a favorable effect. Nine of these interventions were found to have an equal or more favorable impact on participants (children or adults) from low-SES backgrounds than participants with high-SES backgrounds (19). The WOC interventions that benefited groups from low SES had in common that they aimed at three or more levels (19). This contrasts with interventions based only on information delivery (individual-level) which are less effective in groups with lower SES when compared to groups from higher SES (12).

One out of the three levels that obesity prevention interventions should include is the society or the school level. Interventions changing either the society- or the school-level environment have benefitted children from socially disadvantaged backgrounds. A systematic review of 41 intervention studies mostly in developed countries observed that interventions that modified a target population's environment were either cost-effective or cost-saving in the long term (73). Some examples of environmental interventions that were either cost-effective or cost-saving in developing and developed countries were the regulation of TV advertisement of unhealthy foods during children TV prime time and the adoption of food labeling as well as the taxation of unhealthy foods and subsidies to encourage healthy eating (73). Specifically, the taxation of sugar-sweetened beverages in some high-income countries improved weight status across different SES groups (74). Besides strategies to improve the society-level environment, policies designed to promote changes in the school environment benefited children from disadvantaged backgrounds (75). For instance, policies aiming to improve school nutritional standards, provide opportunities for physical activity, as well as deliver nutritional and physical activity education improved the anthropometric measures, diet quality, and physical activity of children with socioeconomic disadvantages (66).

Environmental interventions have beneficial effects across SES groups, but environmental interventions by themselves may not prevent obesity in children. Environmental interventions have the advantage that they address structural barriers, but there may be other specific barriers within the school or the community level of the children and their families that need to be considered. Some researchers have explained that environmental interventions by themselves can widen health disparities because they are delivered equally to all groups, and therefore, they favor socioeconomic groups with more resources to change behaviors (76). Thus, environmental interventions should be accompanied with other strategies that involve the children and their families like the FLVS study. The FLVS study is an example of an intervention that reached many levels (individual-, household-, school-, community-, and society-level) for a 12-year period and that reduced the prevalence of obesity in children from disadvantaged backgrounds (70).

In addition to interventions targeting various levels related to obesity, successful multilevel interventions in low-SES groups are characterized by community collaboration and the participation of actors from different levels and for being sustainable over time (12, 77). The majority of multilevel interventions that have benefited participants from low-SES groups had community engagement in common (19). Community engagement and participation of different actors are characteristics present in the FLVS study. Both characteristics aided the intervention to be sustainable over time. Based on the experience with the FLVS study, researchers developed the EPODE approach. The EPODE is a capacity-building approach for communities to implement effective strategies to prevent obesity in children (78). Besides community participation and engagement, the EPODE approach encourages the involvement of actors or stakeholders from the private and public sector to promote healthy lifestyles (78). Actors are the families, teachers, health professionals, community leaders, and representatives from the private sector and from non-governmental and governmental organizations who can promote healthy behaviors and, consequently, a healthy weight status in children. Under the EPODE approach, multilevel interventions should be sustainable over time, culturally and socially sensitive, and tailored to the needs of all socioeconomic groups (78).

## Final Comments

Despite the inherent limitation of this review (lacking of systematic literature search), the present review synthetized the pertinent literature related to social disadvantage and its consequences for behaviors that could lead to obesity in children. Obesity etiology is multifactorial and therefore complex to explain. To this date, there is no sufficient literature that shows causality between social disadvantages and obesity, but social disadvantages are associated with obesity and obesogenic behaviors. Previous publications have identified that social and economic disadvantages can influence the likelihood of obesity as early as in the conception stages.

SEMs illustrates that a single determinant by itself does not result in obesity in children but that obesity is the result of the complex relationship and interrelationship of various determinants within the individual, family, school, community, and society-levels. Those factors and their relationships can change over time and could be different according to the population (characteristics and place of living) and the social disadvantages present at the individual, family, school, community, and society levels. The best approach to tackle obesity in children from disadvantaged backgrounds is to implement multilevel strategies taking into consideration the social and economic disadvantages. Multilevel interventions allow simultaneously addressing determinants associated with obesity from various levels. There is no “one size fits all” multilevel intervention that could globally prevent obesity. The differences within communities, within and across countries may account for different approaches to prevent obesity. Using frameworks that combine the cumulated research knowledge about obesity prevention and quantitative and qualitative data from the community can help establish the prevention priorities according to the community and country setting. Studies have found that interventions that effectively reduce weight and obesogenic behaviors in participants from disadvantaged backgrounds share four qualities: (1) address at least three levels of influence, (2) the presence of community participation and engagement, (3) the involvement of multi-sectorial stakeholders, and (4) sustainability over time. Multilevel interventions should include a combination of policies that address structural barriers (at the school, community or society-level) and strategies involving the individual and their family. Community engagement is a facilitator in the development of culturally sensitive interventions that address economic disadvantages in a specific community. Also, community engagement is crucial for the implementation and sustainability of interventions. Effective multilevel strategies that help to reduce social and health disparities will represent an economic relief in the costs of obesity and in the costs of chronic diseases associated with obesogenic behaviors.

## Author Contributions

AA-M conceptualized the manuscript. AA-M and II drafted the manuscript. All authors revised and approved the final manuscript.

## Conflict of Interest

The authors declare that the research was conducted in the absence of any commercial or financial relationships that could be construed as a potential conflict of interest.
